# Modeling of a Single-Notch Microfiber Coupler for High-Sensitivity and Low Detection-Limit Refractive Index Sensing

**DOI:** 10.3390/s16050672

**Published:** 2016-05-11

**Authors:** Jiali Zhang, Lei Shi, Song Zhu, Xinbiao Xu, Xinliang Zhang

**Affiliations:** Wuhan National Laboratory for Optoelectronics, Huazhong University of Science and Technology, Wuhan 430074, China; Janny@hust.edu.cn (J.Z.); hustzhusong@hust.edu.cn (S.Z.); xbxu@hust.edu.cn (X.X.); xlzhang@mail.hust.edu.cn (X.Z.)

**Keywords:** optical microfiber sensors, asymmetric coupler, low detection limit

## Abstract

A highly sensitive refractive index sensor with low detection limit based on an asymmetric optical microfiber coupler is proposed. It is composed of a silica optical microfiber and an As_2_Se_3_ optical microfiber. Due to the asymmetry of the microfiber materials, a single-notch transmission spectrum is demonstrated by the large refractive index difference between the two optical microfibers. Compared with the symmetric coupler, the bandwidth of the asymmetric structure is over one order of magnitude narrower than that of the former. Therefore, the asymmetric optical microfiber coupler based sensor can reach over one order of magnitude smaller detection limit, which is defined as the minimal detectable refractive index change caused by the surrounding analyte. With the advantage of large evanescent field, the results also show that a sensitivity of up to 3212 nm per refractive index unit with a bandwidth of 12 nm is achieved with the asymmetric optical microfiber coupler. Furthermore, a maximum sensitivity of 4549 nm per refractive index unit can be reached while the radii of the silica optical microfiber and As_2_Se_3_ optical microfiber are 0.5 μm and a 0.128 μm, respectively. This sensor component may have important potential for low detection-limit physical and biochemical sensing applications.

## 1. Introduction

Evanescent-field based optical sensors have been widely studied in various sensing applications [1,2,3,4]. These sensors have plenty of great properties such as high sensitivity, immunity to electromagnetic interference, and fast response. Among all these sensors based on evanescent field, optical microfiber (OMF) structures have been proved to have good prospects because of their low transmission loss, large evanescent field and small size [5,6,7,8]. With the character of high diameter uniformity and low surface roughness of fabricated optical fiber taper, optical fiber can guide light with low-loss [9,10,11]. Due to its small size, the OMF has a large evanescent field outside of the fiber which makes it sensitive to the index change of the surrounding medium [12,13]. Here, the wavelength shift of the transmission spectrum is often used to characterize the refractive index change caused by the index change of the surrounding medium. These structures based on OMFs include directional couplers [14,15], loop resonators [16,17,18,19], coils [20], Mach-Zehnder interferometers [13] and photonic crystal fiber couplers [21,22], *etc*. For the coil resonator sensor, the detection limit can approach 10^−7^ and the sensitivity is 700 nm per refractive index unit (RIU) with the nanofiber radius of 300 nm [23,24]. Moreover, the supported microfiber loops for optical sensing have been estimated that the detection limit can reach 1.8 × 10^−5^ and the sensitivity is 109.7 nm/RIU [18]. Recently, for the sensor based on the OMF coupler, a sensitivity of 4155 nm/RIU has been achieved [14]. It demonstrates that the sensitivity of the OMF couplers is higher than that of other complicated structures such as loop resonators and coils.

In this paper, we propose and design an asymmetric OMF coupler composed of silica and As_2_Se_3_ for optical sensing. The asymmetric structure here is in order to realize that the transmission spectrum of the coupler has only one dip at the whole single-mode propagation band. Therefore, the detection mechanism is easier than that of the symmetric structure. Meanwhile, the refractive indices of silica and As_2_Se_3_ are 1.444 and 2.83 at 1.55 μm, respectively. Due to the large refractivity index difference, the asymmetric coupler obviously compresses the bandwidth compared with the symmetry structure. Therefore, the sensor based on the asymmetric coupler can reach a lower detection limit which means a smaller change of environmental refractive index can be detected [25].

In our work, the concentration of the surrounding solution is determined by measuring the wavelength shift of the transmission spectrum of the coupler. We take advantage of the important characteristics of the OMF which has the large evanescent field to achieve high-sensitivity sensing. Moreover, we used the feature of the asymmetric structure to achieve a single notch and a narrow bandwidth which can lower the detection limit. In all the simulations, a 3-D finite-difference time-domain (FDTD) method has been used to obtain the light propagation in the coupler [26,27]. By analyzing and discussing the OMF diameters and the distance between the two OMFs, we demonstrate that the sensitivity of the sensor can reach 3212 nm/RIU with a bandwidth of 12 nm and a coupling length of 406 μm. Besides, a maximum sensitivity of this sensor is about 4549 nm/RIU with a bandwidth of 228 nm while the radii of the silica and As_2_Se_3_ OMFs are 0.5 μm and a 0.128 μm, respectively.

## 2. Sensor Principle

**Figure 1:**
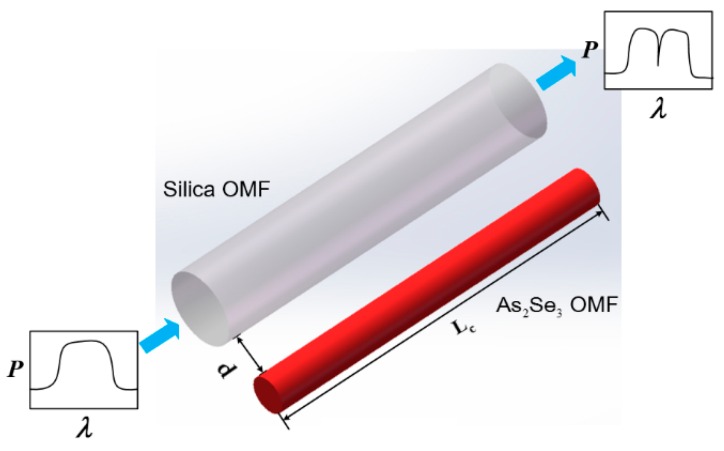
The structure of the sensor component.

The structure of the sensor component is shown in Figure 1. This sensor component is based on the coupling of asymmetric OMFs, which are composed of silica and As_2_Se_3_. R_1_ and R_2_ are the radii of the silica and the As_2_Se_3_ OMFs, respectively. d is the distance between the surface of the two microfibers. L_C_ is the length of the asymmetric OMF coupler.

According to the coupled mode theory, the coupling efficient of the optical power in port to the out port can be expressed as [28]

(1) $$\eta =1-{\left(\kappa L\right)}^{2}{\left[\frac{\sin\sqrt{{\left(\kappa L\right)}^{2}+{\left(\delta L\right)}^{2}}}{\sqrt{{\left(\kappa L\right)}^{2}+{\left(\delta L\right)}^{2}}}\right]}^{2}$$

where κ = (β_+_ − β_−_)/2 is the mode coupling coefficient. β_+_ and β_−_ are the propagation constants of the odd supermode and the even supermode of the asymmetric coupler, respectively. L_C_ = π/2κ is the coupling length, and the phase-mismatch factor δ is defined as

(2) $$\delta =\frac{\pi \left(n_{\mathrm{eff}1}-n_{\mathrm{eff}2}\right)}{\lambda }$$

where n_eff1_ and n_eff2_ are the effective refractive index of the guided mode of the silica OMF and the As_2_Se_3_ OMF, respectively. Due to the asymmetry of the two OMFs, the transmission spectrum of the sensor has only one resonant dip and there is a certain coupling length for the coupler. Based on Equation (1), the full-width at half-maximum (FWHM) bandwidth of the asymmetric OMF coupler at the out port can be expressed as [29]

(3) $$\Delta \lambda =\frac{2\lambda^{2}\kappa }{\pi \left(n_{g1}-n_{g2}\right)}\sqrt{{\left(\frac{2m+2}{2m+1}\right)}^{2}-1}$$

where n_g1_ and n_g2_ are the modal group indices of the two OMFs with m = 0, 1, 2,… According to Equation (3), the bandwidth of the transmission spectrum is inversely proportional to the modal group index difference. Therefore, if the two OMFs are asymmetric, the bandwidth can be obviously compressed.

The sensor component is surrounded with an aqueous medium where analyte exists. The variation of the analyte concentration changes the refractive index of the surrounding aqueous medium. Such a variation of the index changes the effective index of the guide mode. Moreover, a shift of the resonant wavelength is induced. The sensitivity of the sensor is defined by [25]

(4) $$S=\frac{\partial \lambda_{c}}{\partial n_{c}}=\frac{\partial \lambda_{c}}{\partial n_{\mathrm{eff}}}\cdot \frac{\partial n_{\mathrm{eff}}}{\partial n_{c}}$$

where n_c_ is the refractive index of the ambient medium, and λ_c_ is the resonant wavelength. According to Equation (4), it can be seen that the sensitivity is divided into two parts. They are called device sensitivity and waveguide sensitivity, respectively. The device sensitivity only depends on the device property, while the waveguide sensitivity depends on the waveguide structure parameter. The two parts separately work on the whole sensitivity. The OMF diameter and the distance between the two OMFs are the main parameters which are directly related to the whole sensitivity. Here, the distance between the two OMFs is the device parameter while the OMF diameter is the waveguide parameter.

Beside the sensitivity, the detection limit is also a key parameter to characterize the sensor. The detection limit is defined as the minimal detectable refractive index change caused by the analyte. Theoretically, the sensitivity and the detection limit are often supposed to be equivalent. However, in an actual sensing experiment, the smallest detectable shift of resonant wavelength δλ_c_ depends on the bandwidth of the transmission dip ∆λ. Therefore, from the experimental point of view, a figure of merit (FOM) of the detection limit can be introduced and defined as [25]

(5) $$\mathrm{FOM}=\frac{\Delta \lambda }{S}$$

where ∆λ is the FWHM bandwidth. Hence, a smaller detection limit can be reached by compressing the bandwidth. Considering the key performance indicators of the sensor are the sensitivity and the detection limit, we discuss the effect of the OMF diameter and the distance between two OMFs on the sensor performance by changing R_1_, R_2_ and d.

Here, the light propagation in the coupler is simulated by the Lumerical FDTD Solutions software. The following results are obtained by the FDTD simulations. Moreover, in order to demonstrate the accuracy of the FDTD simulations, analytical simulations are also presented in the following results which are obtained by solving Equation (1). Considering the factors influencing the sensor performances, we mainly carry on the analysis and discussion on R_1_ and d. Because R_2_ varies with R_1_, we only discuss the effect of R_1_ on the sensor performance instead of the two radii. n_c_ is 1.318 in water at 1.55 μm wavelength (25 °C). In order to guarantee the single-mode propagation, R_1_ varies from 0.5 to 0.9 μm. We change the distance from 500 nm to 2000 nm, while the surrounding aqueous medium n_c_ varies from 1.318 to 1.328. Phase match wavelength is set at 1.55 μm.

## 3. Results and Discussion

**Figure 2:**
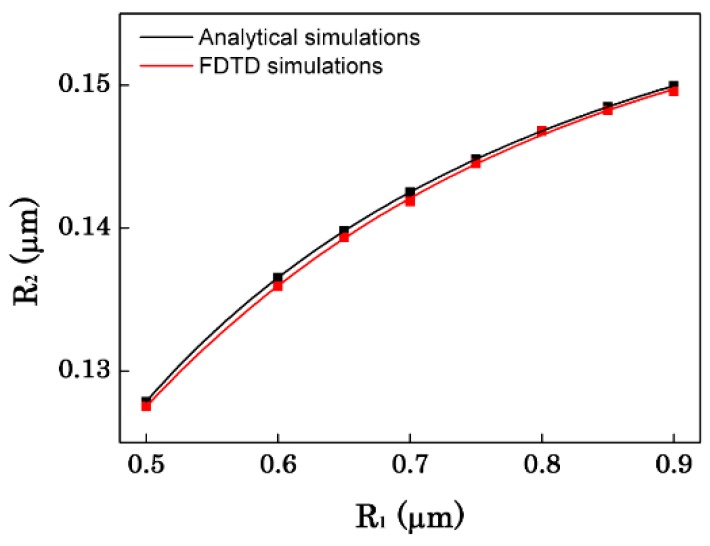
The optical microfiber (OMF) radius of phase match at λ = 1.55 μm.

**Figure 3:**
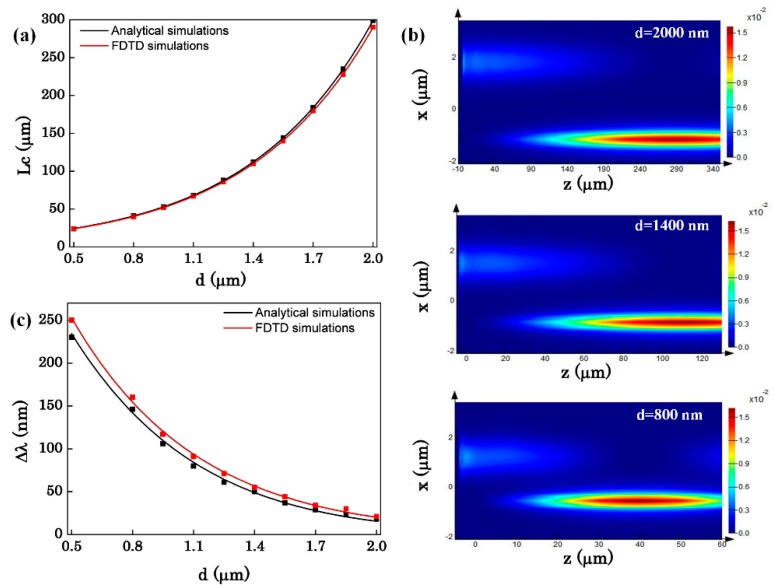
(**a**) The coupling length as a function of the distance between two OMFs; (**b**) The power maps of the evanescent coupling between silica OMF and As_2_Se_3_ OMF with d = 2000, 1400, 800 nm; (**c**) The bandwidth of the transmission spectrum as a function of the distance between two OMFs (R_1_ = 0.8 μm, R_2_ = 0.147 μm, n_c_ = 1.318).

First of all, we discuss the effect of the distance between the two OMFs on the sensor performance. Figure 2 represents the corresponding phase match radius of the two OMFs at the 1.55 μm wavelength. It is shown that R_1_ and R_2_ are corresponding increasing in order to complete coupling. Figure 3 shows the influence of distance (d) between the two OMFs to the coupling length, the power maps of the coupling between the two OMFs, and the bandwidth of the transmission spectra. As can be seen in Figure 3a, the sensor length increases with the increase of the distance d. That can be explained by the coupled mode theory. When the two OMFs are close to each other, the fundamental mode in one fiber will couple to the other fiber. Conversely, when the distance of the two OMFs increase, it will become more difficult to light power coupling. Figure 3b gives the power maps with different distances between the two OMFs. From Figure 3b, we can see more intuitively that the farther the distance of the two OMFs, the longer the coupling length. Meanwhile, the distance also affects the bandwidth. Figure 3c represents the variation of the bandwidth with the distance. As shown in Figure 3c, ∆λ decreases as d increases. Because of the large evanescent wave outside the fiber, when the two OMFs are approached, the coupling of phase mismatch wavelength can also be significantly enhanced. Therefore, the closer the two OMFs are, the wider the bandwidth is.

**Figure 4:**
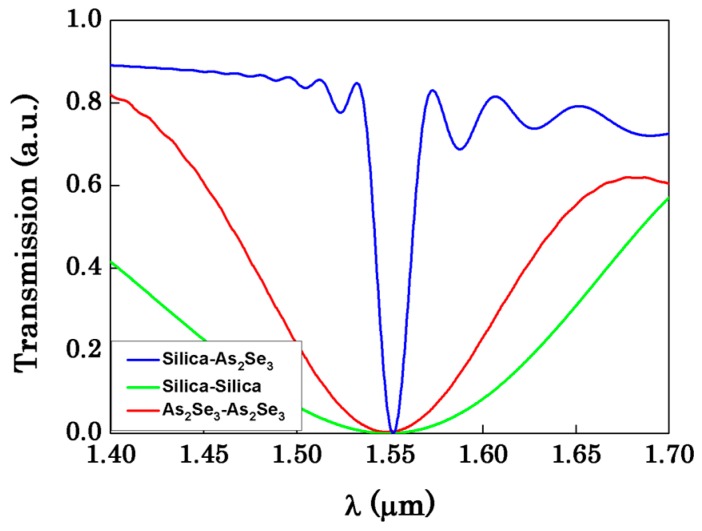
Transmission spectra of the asymmetric OMF coupler and the symmetric OMF coupler, R_1_ = 0.8 μm, R_2_ = 0.147 μm, d = 2000 nm.

Then, we verify the single-notch and narrowband characteristics of the asymmetric coupler. As can be seen in Figure 4, there is only one resonant dip at the wavelength ranging from 1.4 μm to 1.7 μm. Due to the asymmetry of the two OMFs, they can only reach complete coupling at a certain wavelength (here is 1.55 μm), so there is only one dip at the whole single-mode band. We can also see that, compared with the symmetric coupler, which is composed of both silica OMFs or As_2_Se_3_ OMFs, the bandwidth of the asymmetric structure is significantly narrower. That is because of the refractive index difference of the asymmetry structure. Here, we just compare with the symmetric coupler composed of both silica OMFs which has been widely studied. The result shows that the bandwidth of our asymmetric structure is about 21 nm, while the bandwidth of silica-silica structure is about 285 nm. It is over one order of magnitude less than the symmetric silica structure. According to Equation (5), the bandwidth of the transmission spectrum is directly related to the detection limit, the larger compressing of the bandwidth will significantly reduce the detection limit. Meanwhile, the sensitivity of our asymmetric structure is 3539 nm/RIU while the sensitivity of silica-silica structure is 4639 nm/RIU. Because the refractive index of silica is 1.444, the refractive index difference between silica and the ambient medium is smaller which make the evanescent field become larger. The larger the evanescent field is, the higher the sensitivity is. So the result also indicates that compared with the symmetric structure, the detection limit of our asymmetric structure could be over one order of magnitude less while the sensitivity is close.

**Figure 5:**
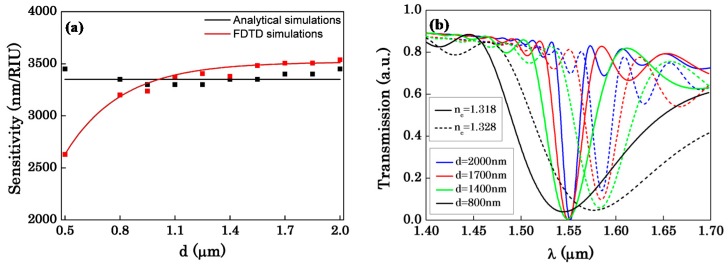
(**a**) The sensitivity as a function of the distance between the two OMFs, R_1_ = 0.8 μm, R_2_ = 0.147 μm; (**b**) The spectrum shift when n_c_ varies from 1.318 to 1.328 for different distances between the two OMFs, R_1_ = 0.8 μm, R_2_ = 0.147 μm.

The effect of d to sensor sensitivity is shown in Figure 5. We can see in Figure 5a, when d is approach to 500 nm, there is a large difference between the analytical simulations and the FDTD simulations. Because Equation (1) is applicable in weak coupling conditions, when the two OMFs become closer the coupling of the two OMFs becomes so strong that Equation (1) is not applicable any more. Generally, the FDTD simulations are more accurate. In addition, we can see intuitively that the sensitivity is almost the same as d increases from the analytical simulations, while the sensitivity increases less and less, eventually keeps flat from the FDTD simulations.

Meanwhile, according to Figure 3c we can also see that, as d increases, the bandwidth of the transmission spectrum significantly decreases. Therefore, we compare the transmission spectrum shift as the change of external environment refractive index (n_c_) with different distances as shown in Figure 5b. We find that the tiny change of the sensitivity is caused by the visible change of the bandwidth. According to Figure 5b, we can see intuitively that there are small changes in the resonant wavelengths of different distances, while the distance varies from 1400 nm to 2000 nm. The sensitivity is decided by the wavelength shift of the transmission spectrum. Therefore, the sensitivity changes relatively slowly. Moreover, we can also see in Figure 5b that as d changes from 1400 nm to 2000 nm, the bandwidth of the transmission spectrum becomes narrower and narrower. In our opinion, the tiny increase of the sensitivity as d increases is due to the significant decrease with the bandwidth, which can also explain why the sensitivity increases less and less even keeps flat as seen in Figure 5a. Hence, in our view, what the distance of the two OMFs changes is the bandwidth. The distance of the two OMFs almost has no influence on the sensitivity. The sensitivity merely is related to the radii of the OMFs. Besides, as can be seen in Figure 5b, when d is 800 nm, the resonant wavelength is shifted to the phase-match wavelength. That is because of the large evanescent field of the OMF, when the two OMFs is approached less than 800 nm, the coupling is so strong that the resonant dip is a little deviation to the phase-match wavelength.

**Figure 6:**
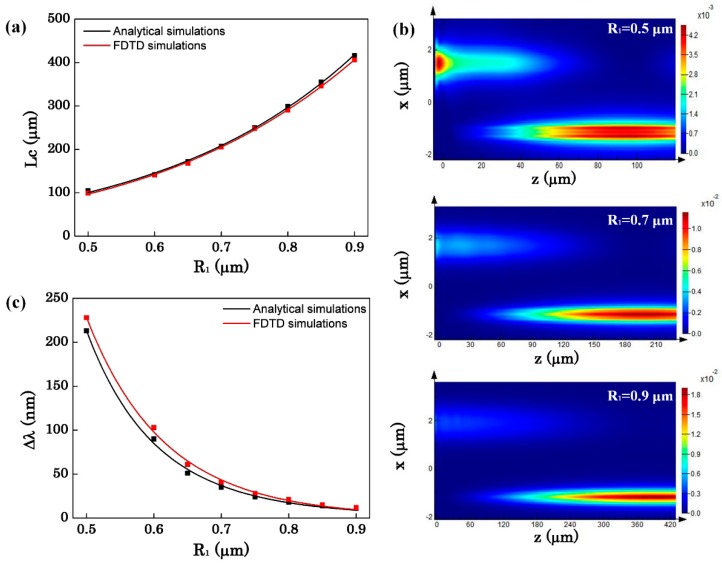
(**a**) The coupling length as a function of the radius of the silica OMF; (**b**) The power maps of the evanescent coupling between the silica OMF and the As_2_Se_3_ OMF with R_1_ = 0.5, 0.7, 0.9 μm, respectively; (**c**) The bandwidth of the transmission spectrum as a function of the radius of the silica OMF with d = 2000 nm.

Then, we discuss the effect of the radii of the two OMFs on the sensor performance. According to the above analysis, we change the radius in the case of d = 2000 nm to achieve a narrow bandwidth. Figure 6a shows the relation between the OMF radius and the coupling length. The coupling length increases while the radius increases because more light is confined in the OMF. The power maps of the evanescent coupling with different OMF radii are shown in Figure 6b. From Figure 6b, we can see that when the radius of the OMF gets larger, more light is confined in the OMF so that the evanescent wave outside the fiber becomes weaker. Furthermore, the weak evanescent wave leads to more difficult coupling which makes the coupling length become longer. Simultaneously, the weaker evanescent wave outside the OMF also causes the narrower bandwidth of the transmission spectrum. Figure 6c illustrates the variation of the bandwidth of the transmission spectrum with the OMF radii. ∆λ increases as R_1_ increases. With the increase of the radius, the confinement of light in the OMFs is enhanced. Then the bandwidth is compressed because the coupling of the two OMFs becomes more difficult.

**Figure 7:**
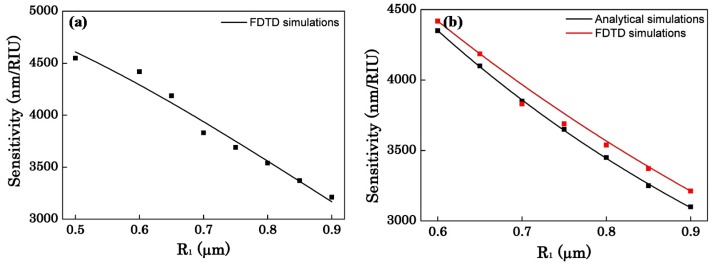
(**a**) The sensitivity as a function of the radius of the silica OMF with d = 2000 nm; (**b**) The sensitivity as a function of the radius of the silica OMF which ranging from 0.6 μm to 0.9 μm with d = 2000 nm.

**Figure 8:**
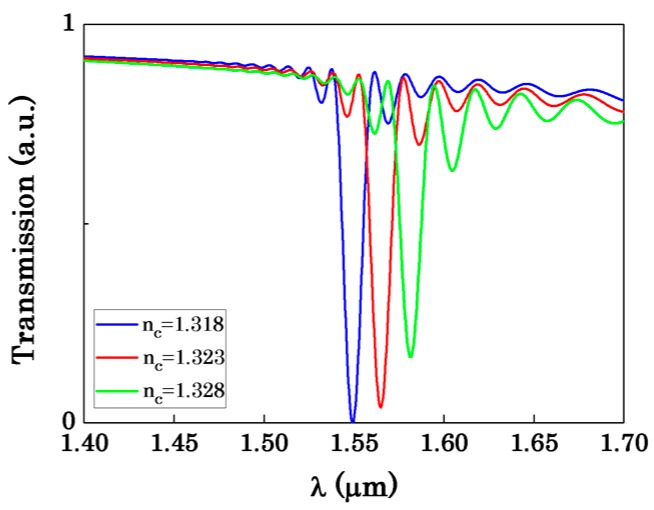
The transmission spectrum with R_1_ = 0.9 μm, R_2_ = 0.15 μm, L_C_ = 406 μm, d = 2000 nm.

After discussing the effect of the radius on the bandwidth, Figure 7 shows the effect of radius on the sensitivity. When R_1_ is small, the coupling of two OMFs is so strong that Equation (1) is not applicable. Therefore, the FDTD simulations are individually presented in Figure 7a with R_1_ ranging from 0.5 μm to 0.9 μm, while the analytical simulations and FDTD simulations are shown in Figure 7b with R_1_ ranging from 0.6 μm to 0.9 μm. From Figure 7b we can see that when it’s weak coupling for the asymmetric coupler, the FDTD simulations and analytical simulations are approximate. Therefore, we just discuss the effect of the radius on the sensitivity obtained by FDTD simulations. From Figure 7a it is seen that as the radius of the OMF increases, the sensitivity of the sensor decreases. Likewise, due to the confinement of light in the MFs, the evanescent field outside the OMF becomes weaker. The change of the evanescent wave will result in that the interaction between light and the surrounding medium becomes weak which reduces the sensitivity. Moreover, as can be seen in Figure 7a, there is a maximum sensitivity of 4549 nm/RIU with R_1_ = 0.5 μm, R_2_ = 0.128 μm, L_C_ = 99 μm and ∆λ = 228 nm. All the results and discussion indicate that when d and R_1_ increase, L_C_ increases and ∆λ decreases. According to Equation (5), ∆λ is proportional to the FOM of the detection limit. With the increase of d, ∆λ decreases and this directly reduces the detection limit. Also with the increase of R_1_, the sensitivity decreases. A trade-off exists between the sensitivity and the bandwidth by choosing suitable waveguide parameters. Figure 8 represents the transmission spectrum shift for the environment refractive index n_c_ changes from 1.318 to 1.328 while R_1_ = 0.9 μm, R_2_ = 0.15 μm, d = 2000 nm. As shown in Figure 8, the sensor sensitivity can reach about 3212 nm/RIU, the bandwidth is about 12 nm and the coupling length L_C_ = 406 μm.

## 4. Conclusions

In summary, a high-sensitivity optical sensor based on the asymmetric optical microfiber coupled structure is proposed. The coupler is composed of silica and As_2_Se_3_ optical microfiber. Lumerical FDTD Solutions is used to simulate the coupling characteristics. The key parameters which affect the sensor performances have been analyzed and discussed. A maximum sensitivity of 4549 nm/RIU can be reached with 0.5-μm silica OMF, 0.128-μm As_2_Se_3_ OMF, and 99-μm coupling length. Also a narrow bandwidth of an asymmetric coupler has been verified. With a trade-off between the sensitivity and the bandwidth, a sensitivity of 3212 nm/RIU with a bandwidth of 12 nm can be achieved. Experimentally, the silica optical microfiber can be fabricated using improved flame-heated technique, and the As_2_Se_3_ optical microfiber can be drawn from bulk As_2_Se_3_-doped glass. After that, they are fixed on two three-dimensional translation stages for easy manipulation. The distance between the two OMFs can be controlled precisely to realize the best sensing performance.
